# Dual regulation of *cueP* by the CueR and Cpx systems enables *Salmonella* adaptation to copper and *N*-chlorotaurine

**DOI:** 10.1093/femsml/uqag018

**Published:** 2026-05-13

**Authors:** Maxence Dessertine, Jérôme Becam, Benjamin Ezraty, Laurent Aussel

**Affiliations:** Aix-Marseille Université, CNRS, Laboratoire de Chimie Bactérienne, UMR7283 Marseille, France; Aix-Marseille Université, CNRS, Laboratoire de Chimie Bactérienne, UMR7283 Marseille, France; Aix-Marseille Université, CNRS, Laboratoire de Chimie Bactérienne, UMR7283 Marseille, France; Aix-Marseille Université, CNRS, Laboratoire de Chimie Bactérienne, UMR7283 Marseille, France

**Keywords:** *Salmonella*, reactive chlorine species, copper homeostasis, adaptive mechanisms, gene regulation, oxidative stress

## Abstract

*Salmonella enterica* is a facultative intracellular pathogen capable of surviving within host cells, where it faces a sophisticated immune arsenal. Within the phagosomal compartment, the bacterium encounters significant stress from copper and reactive chlorine species like *N*-chlorotaurine (*N*-ChT) generated during the oxidative burst. We investigated the regulatory mechanisms enabling *Salmonella* to adapt to this dual copper/oxidative stress, specifically focusing on the regulation and function of CueP. This periplasmic protein was previously proposed to bind copper ions and to transfer them to the superoxide dismutase SodCII. Here, we demonstrated that copper specifically triggered the CueR pathway and that *N*-ChT activated the Cpx pathway; simultaneous exposure to both stresses resulted in maximum *cueP* expression levels. Moreover, CueP was shown to be important for copper resistance in the absence of the multicopper oxidase CueO and exhibits high thermostability in the presence of copper. Additionally, in a Δ*cueO* background, copper is sufficient to activate the Cpx pathway, ensuring robust *cueP* induction even without external oxidative signals. These findings establish a direct molecular link between a host antimicrobial agent (*N*-ChT) and the activation of the Cpx-CueP axis, revealing a new layer of bacterial adaptation to innate immunity. Moreover, they highlight an integrated response strategy contributing to bacterial adaptation to the dual copper/oxidative stress.

## Introduction

To combat invading pathogens, immune cells deploy a refined antibacterial arsenal encompassing various stressors, including reactive oxygen species (ROS), reactive chlorine species (RCS), acidification of the phagosomal environment, metal toxicity, antimicrobial peptide production, or nutrient deprivation. Copper (Cu), an essential transition metal, is required for numerous bacterial enzymatic processes; however, its redox properties confer cytotoxicity at elevated concentrations. Under both aerobic and anaerobic conditions, copper toxicity manifests through (i) oxidation of thiol groups, (ii) displacement of other transition metals from metalloproteins, and (iii) protein aggregation, ultimately impairing bacterial viability (Checa et al. 2021). Copper commonly exists in two redox states: Cu⁺ and Cu²⁺. In the highly reducing environment of the cytoplasm, Cu⁺ is the predominant form, whereas both redox states coexist within the more oxidising conditions of the periplasm. To maintain copper homeostasis, bacteria have evolved intricate regulatory networks that coordinate copper acquisition, utilisation, and detoxification (Ladomersky and Petris 2015). In *Salmonella enterica*, a facultative intracellular pathogen, copper homeostasis is critical for survival in diverse environmental and host-associated niches, particularly within the phagosomal compartment, where copper concentrations increase as part of the host immune response (Checa et al. 2021, Espariz et al. 2007, Achard et al. 2012, Zhao et al. 2026). To counteract copper toxicity, *Salmonella* require detoxification systems. CopA functions as an inner membrane ATPase that pumps copper from the cytoplasm into the periplasm while CueO is a periplasmic multicopper oxidase (Checa et al. 2021). CueP was proposed to act as a periplasmic cuprotein that accepts copper ions from CopA and deliver them to the periplasmic Cu/Zn-superoxide dismutases SodCI and SodCII (Osman et al. 2013, Fenlon and Slauch 2017). Consistently, *copA, cueO*, and *cueP* are under the control of the same transcriptional regulator, CueR, previously shown to act as a cytoplasmic Cu^+^ sensor (Espariz et al. 2007, Pontel and Soncini 2009, Achard et al. 2010).

In addition to metal toxicity, immune cells exert antimicrobial pressure through the generation of highly reactive oxidants. This process, broadly termed the “oxidative burst,” results in the production of a range of ROS, including superoxide anions (O₂•⁻), hydrogen peroxide (H₂O₂), and hydroxyl radicals (HO•), as well as RCS such as hypochlorous acid (HOCl) and *N*-chlorotaurine (*N*-ChT). These oxidants inflict extensive damage on bacterial macromolecules, including DNA, proteins, and lipids, culminating in cell death. To counteract these cytotoxic effects, *S. enterica* has evolved a set of defense mechanisms, including antioxidant enzymes that reduce or degrade ROS, thereby mitigating oxidative stress and enhancing bacterial survival within the host (Imlay 2019, Rhen 2019, Hébrard et al. 2009).

One of the components of *S. enterica* copper homeostasis is the *cueP* gene, which encodes the CueP periplasmic protein. *cueP* is a constituent of the CueR regulon, a transcriptional regulator that responds to elevated cytoplasmic copper levels, thereby orchestrating an adaptive response to metal-induced stress (Pontel and Soncini 2009). In addition, the Cpx two-component system, which predominantly detects envelope stress, has also been shown to be involved in the regulation of *cueP* expression (Pezza et al. 2016). CpxR regulates many genes encoding proteins maintaining cell wall integrity, such as the chaperones CpxP and Spy, or the methionine sulfoxide reductase MsrP (Yamamoto and Ishihama 2006, Appia-Ayme et al. 2012, Andrieu et al. 2023). CpxR was also proposed to modulate *cueP* transcription in response to membrane stress, thereby facilitating *S. enterica* adaptation to the hostile intracellular environment of the host (Pezza et al. 2016). The integration of the CueR and CpxR sensory systems ensures that the production of this periplasmic protein is optimised in cells simultaneously experiencing copper excess and membrane stress.

In most enteric species, periplasmic copper homeostasis is mediated by the CusCFBA efflux system (Franke et al. 2003). However, *Salmonella* species have lost these genes from their core genomes and instead acquired *cueP*, highlighting its potential role in adaptation to an intracellular lifestyle (Checa et al. 2021, Méndez et al. 2022). Results on the role of copper in *S. enterica* virulence have been somewhat variable. Competitive assays in mice conducted by different groups have shown that the *copA, golT, cueO*, and *cueP* genes were dispensable during systemic infection (Fenlon and Slauch 2017, Cunrath and Bumann 2019). In contrast, evidence suggests that CueP contributes to bacterial survival within macrophages, where it may alleviate copper toxicity encountered in the phagosomal compartment (Yoon et al. 2014). Structural analyses indicate that CueP possesses cysteine and histidine residues involved in copper coordination, which likely facilitate its role in copper homeostasis (Yoon et al. 2013). CueP has also been shown to contribute to SodC metalation, suggesting a role in periplasmic oxidative stress management (Osman et al. 2013, Fenlon and Slauch 2017). Furthermore, ScsB and ScsC were found to bind Cu^+^ with high affinity and transfer it to CueP (Subedi et al. 2019). However, the precise biochemical mechanisms underlying CueP function, including potential copper scavenging or chaperone-like activities, remain incompletely characterised.

Our recent findings have demonstrated that *N*-ChT activates the Cpx pathway, thereby establishing a connection between oxidative stress and the envelope stress response (Andrieu et al. 2023). In the present study, we provide evidence that *cueP* is simultaneously regulated by both copper and *N*-ChT. Furthermore, we show that CueP plays an important role in copper resistance in the absence of CueO. Finally, we demonstrate that the presence of copper increases the stability of CueP, which could contribute to *Salmonella*’s adaptation to copper-rich environments.

## Results

### The CueR pathway is specifically activated by copper, and the Cpx pathway by *N*-chlorotaurine

**Figure 1:**
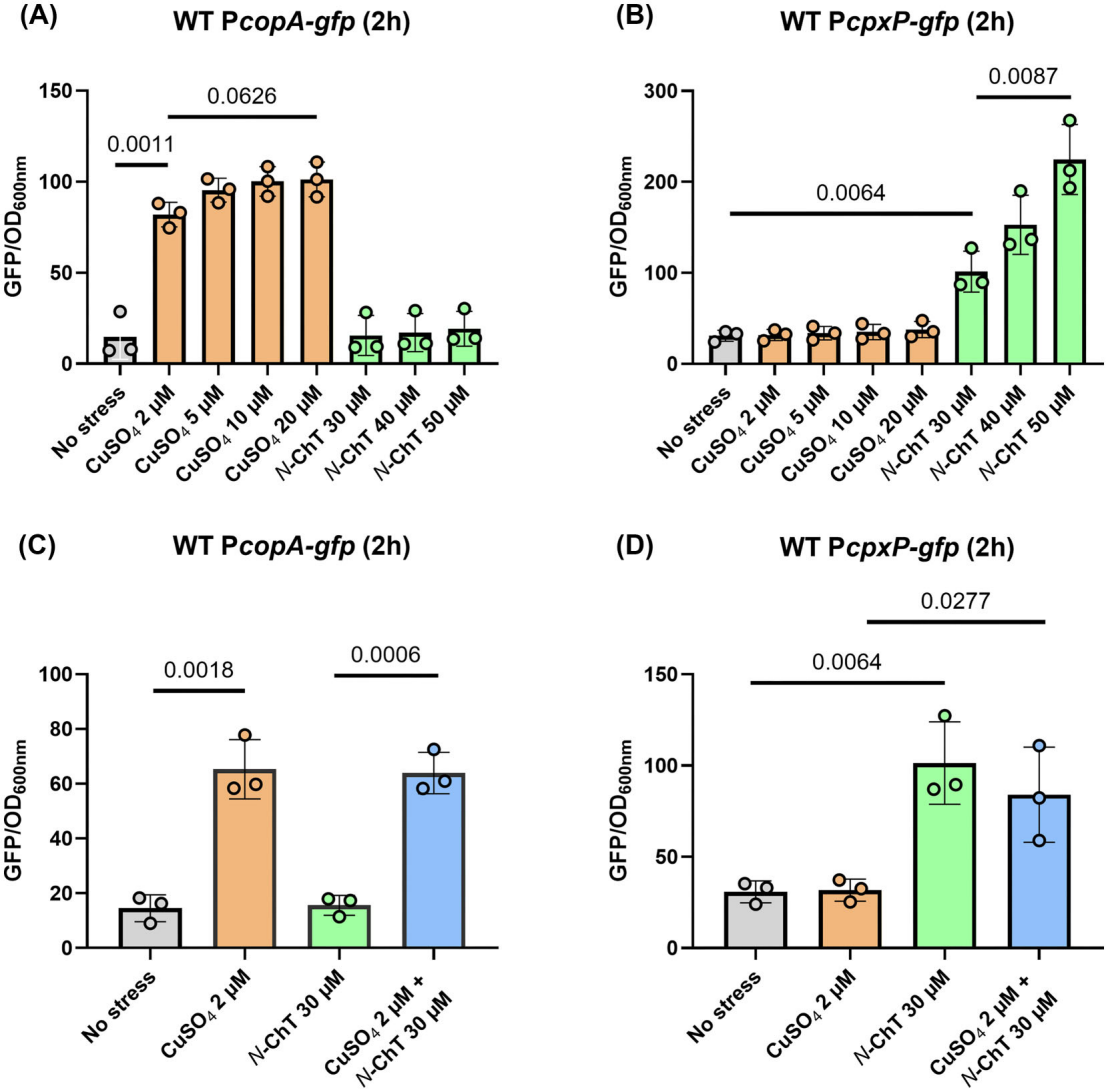
Activation of the CueR and Cpx pathways by copper and *N*-chlorotaurine. A WT *Salmonella enterica* strain carrying a P*copA-gfp* fusion (A and C) or a P*cpxP-gfp* fusion (B and D) was grown aerobically in M9 medium. At OD_600_ = 0.3, the strains were subjected to copper stress (orange bars), *N*-ChT stress (green bars), dual copper/*N*-ChT stress (blue bars), or no stress (grey bar). The fluorescence of the fusions was measured using a microplate reader and normalised to the OD_600_ 2 h post-stress. Results are the means ± standard deviation of three independent experiments. Statistical analysis was performed using Student’s *t*-test. Exact *P*-values are reported for comparisons with *P* < 0.05 considered statistically significant.

The regulation of the *cueP* gene involves two distinct regulatory pathways: the CueR and Cpx pathways. However, when this model was initially proposed, the stimulus activating the Cpx pathway remained unknown. We recently demonstrated that *N*-ChT triggers the Cpx pathway, and we now aim to elucidate the role of this stimulus in *cueP* regulation. To investigate this, we constructed two transcriptional fusions whose expression reflects the activation of the CueR and Cpx pathways: P*copA-gfp* and P*cpxP-gfp*, respectively. Upon exposure to increasing concentrations of copper, we observed a marked increase in fluorescence from the P*copA-gfp* fusion, whereas the addition of *N*-ChT had no effect (Fig. 1A). Fluorescence monitoring over time revealed a very rapid increase 2 h after copper stress, followed by sustained dose-dependent activation over time (Fig. S1A). Furthermore, this activation is exclusively dependent on the CueR regulator, as no induction of P*copA-GFP* fusion was observed in a Δ*cueR* mutant in response to increasing doses of copper, compared with a wild-type (WT) strain (Fig. S1B). Conversely, copper did not enhance fluorescence in the P*cpxP-gfp* fusion, while increasing doses of *N*-ChT in the culture medium led to a proportional increase in fluorescence from this reporter (Fig. 1B). Here again, expression of the P*cpxP-gfp* fusion peaks 2 h after *N*-ChT treatment, followed by a sustained level proportional to the dose of *N*-ChT over time (Fig. S1C). The P*cpxP-gfp* fusion was finally tested in a Δ*cpxR* mutant in response to increasing doses of *N*-ChT, and no activation was detected, unlike in the WT strain (Fig. S1D).

Next, we selected 2-µM copper, which induces the CueR pathway without affecting Cpx, and 30-µM *N*-ChT, which activates the Cpx pathway without influencing CueR. Under these conditions, we observed that copper alone and the combined copper/*N-*ChT stress activated the P*copA-gfp* fusion to the same extent (Figs 1C and S1E). Consistently, *N*-ChT alone and the combined copper/*N*-ChT stress induced P*cpxP-gfp* fluorescence at comparable levels (Figs 1D and S1F). These results confirm that the CueR and Cpx pathways are specifically activated by copper and *N*-ChT stress, respectively. They also show an absence of cross-activation between these stimuli and their signaling pathways using copper concentrations up to 30-µM and *N*-ChT concentrations up to 50-µM.

### *cueP* is highly expressed after simultaneous copper/*N*-ChT stress

**Figure 2:**
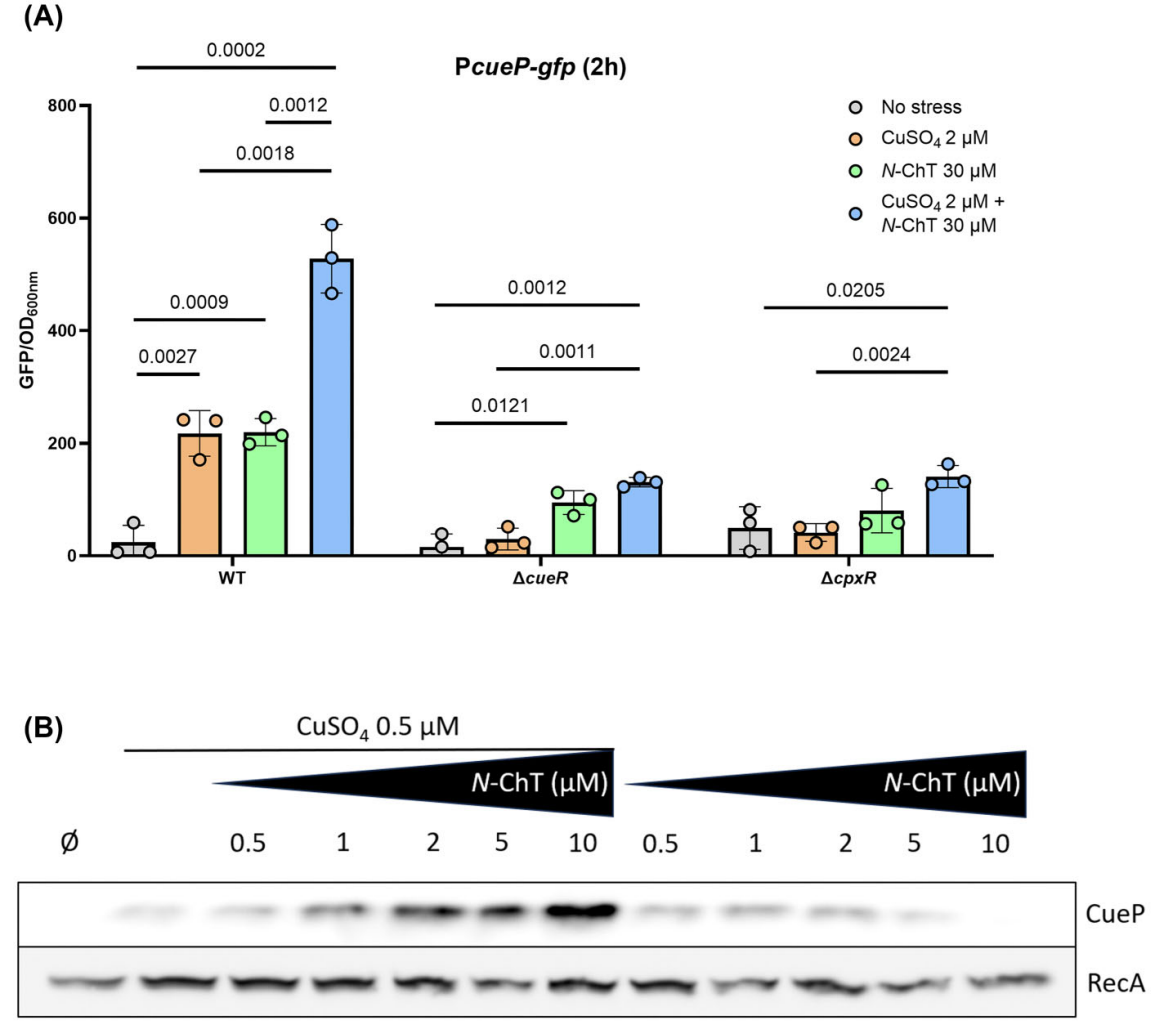
*cueP* expression is activated by copper/*N*-chlorotaurine dual stress. (A) A WT strain, a Δ*cueR* mutant, and a Δ*cpxR* mutant carrying a P*cueP-gfp* fusion were grown aerobically in M9 medium. At OD_600_ = 0.3, the strains were subjected to copper stress (orange bars), *N*-ChT stress (green bars), dual copper/*N*-ChT stress (blue bars), or no stress (grey bar). The fluorescence of the fusions was measured using a microplate reader and normalised to the OD_600_ 2 h post-stress. Statistical analysis was performed using Student’s *t*-test. Exact P-values are reported for comparisons with *P* < 0.05 considered statistically significant. (B) *Salmonella enterica* cells producing CueP-SPA were grown in M9 minimal medium at 37°C. At an OD_600_ of 0.1, cells remained untreated (lane 1), treated with CuSO_4_ 0.5 µM (lane 2), treated with increasing concentrations of *N*-ChT (0.5–10 µM) in the presence (lanes 3–7) or the absence (lanes 8–12) of CuSO_4_ 0.5 µM for 1 h. CueP proteins were analyzed by immunoblotting. RecA was used as an internal loading control, stained on the same blot. These blots are representative of three independent experiments.

We then tested a minimum concentration of copper, specifically activating the CueR pathway (2 µM), and a minimum concentration of *N*-ChT, specifically activating the Cpx pathway (30 µM), on the P*cueP-gfp* fusion. Interestingly, we found that *cueP* was partially activated in response to either copper or *N-*ChT (9-fold), whereas the combined stress led to a more pronounced activation (22-fold) (Fig. 2A, left). Monitoring of *cueP* expression over time shows a rapid increase in fluorescence in the two h after dual stress, followed by a slower but constant slope up to 10 h post-stress (Fig. S1G). Next, we sought to evaluate the contribution of the CueR and Cpx pathways to *cueP* expression under dual stress conditions. To this end, we compared the expression of the P*cueP-gfp* fusion in a WT strain, a Δ*cueR* mutant, and a Δ*cpxR* mutant. In both mutants, *cueP* expression in response to the dual stress was markedly reduced compared to the WT (WT) strain (Fig. 2A, right). To strengthen these results, we carried out a Western blot analysis to examine the production of CueP in the periplasm. To this end, we introduced a SPA-tag at the *cueP* locus and assessed CueP production one hour after stress exposure. No detectable signal was observed under unstressed conditions, whereas a faint band appeared following exposure to 0.5-µM copper (Fig. 2B). When increasing concentrations of *N*-ChT were added to the medium containing 0.5-µM Cu, band intensity increased proportionally to *N*-ChT concentration, confirming that CueP production is regulated by this dual stress. Finally, when applying an *N*-ChT gradient in the absence of copper, we detected a weak CueP signal, which gradually decreased (Fig. 2B). This decline can be attributed to the absence of copper—which post-translationally stabilises CueP—coupled with the oxidative properties of *N*-ChT, which exert the opposite effect. These results are consistent with previous findings (Pezza et al. 2016) and unexpectedly demonstrate that *Salmonella* is capable of integrating two different environmental stimuli, which in turn activate two regulatory pathways to trigger the expression of *cueP*. They also suggest that the CueR and Cpx pathways act additively, yet the loss of either one reduces *cueP* expression under all conditions.

### CueP plays an important role in copper resistance

**Figure 3:**
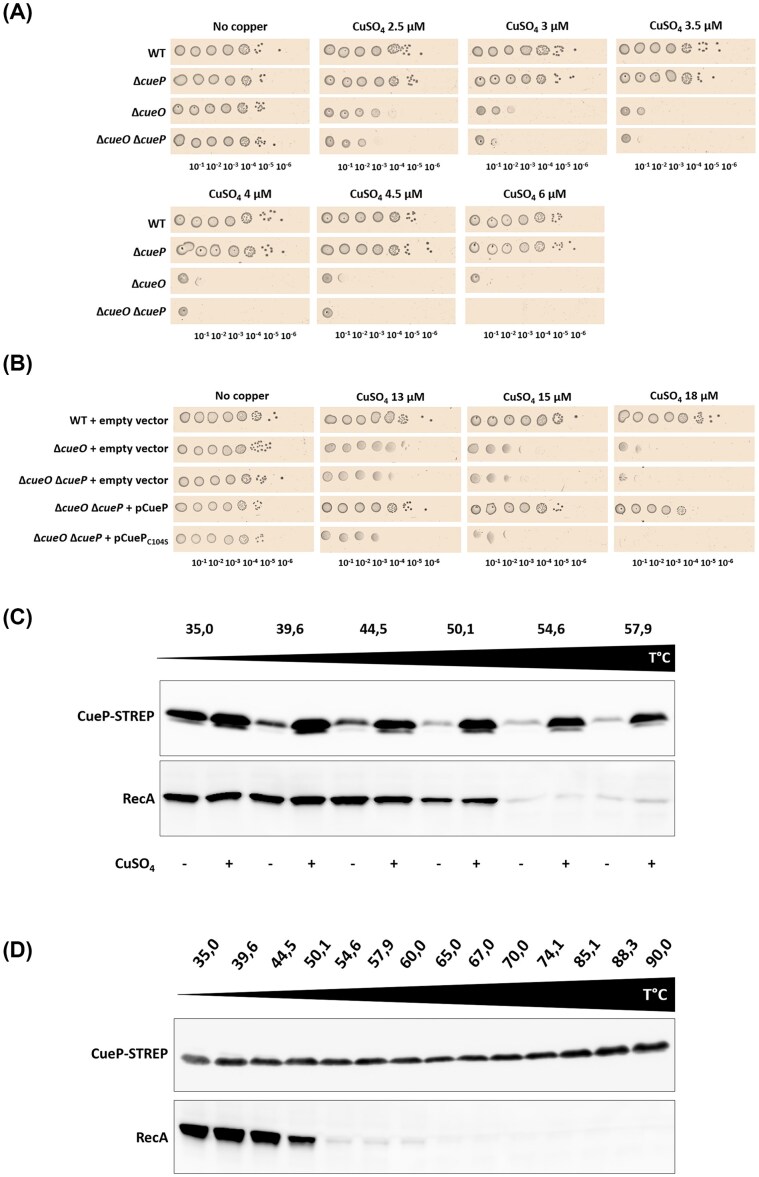
CueP is involved in copper resistance in *Salmonella*. (A) Plating efficiency of WT, Δ*cueP*, Δ*cueO*, and Δ*cueO* Δ*cueP* strains in the presence of CuSO_4_. All the strains wells were grown in M9-glucose medium under aerobic conditions at 37°C. At an OD_600_ of 0.1, cells were diluted and 10-fold serial dilutions were spotted onto M9-glucose plates, with or without the addition of CuSO_4_ at the concentrations given (top panel). Plates were incubated for two days and then scanned. This spotting experiment is representative of at least three independent experiments. (B) WT and Δ*cueO* strains carrying an empty vector (pJF119EH), and Δ*cueO* Δ*cueP* strains carrying an empty vector or pCueP plasmids, which overproduced CueP, were grown in M9-glucose medium, supplemented with ampicillin and IPTG, under aerobic conditions at 37°C. At an OD_600_ of 0.1, the procedure described in (A) was followed. M9-glucose plates supplemented with ampicillin (100 μg/mL), IPTG (100 μM), and copper were used. This spotting experiment is representative of three independent experiments. (C–D) Cellular thermal shift assay of CueP fused to STREP-Tag. The experiments were carried out in WT cells with (+) or without (−) 50-µM CuSO_4_ (C), and with 50-µM CuSO_4_ (D). Aliquots were subjected to thermal gradients for 3 min. Cells were lysed, centrifuged, and the supernatants filtered to remove protein aggregates. CueP was detected using HRP-conjugated anti-STREP antibody. As a loading control, run on the same gel, anti-RecA antibodies and HRP-conjugated anti-rabbit secondary antibodies were used. These blots are representative of three independent experiments.

To better understand the role of CueP in copper resistance in *S. enterica*, we compared the sensitivity of a Δ*cueP* mutant to that of a WT strain on agar plates containing increasing doses of copper. Both strains showed the same viability in response to this metal stress (Fig. 3A). We hypothesised that the role of CueP may have been masked by another periplasmic protein involved in copper homeostasis. To test this idea, we evaluated the resistance of a Δ*cueO* mutant lacking multicopper oxidase activity and therefore unable to oxidise periplasmic Cu⁺ to Cu²⁺. As previously demonstrated, the Δ*cueO* mutant exhibits increased sensitivity to copper (Fig. 3A) (Pontel and Soncini 2009, Achard et al. 2010). When the two mutations are combined (Δ*cueO* Δ*cueP*), this mutant is slightly more sensitive to copper than the Δ*cueO* mutant, revealing a role for CueP in copper homeostasis (Fig. 3A). This observation is strengthened by the fact that a Δ*cueO* Δ*cueP* mutant carrying a pCueP plasmid shows copper resistance equivalent to that of a WT strain carrying an empty plasmid (Fig. 3B). This result is consistent with previous data showing that *cueP* overexpression in a Δ*cueO* Δ*cueP* double mutant (formerly known as Δ*cuiD* Δ*cueP*) increased the resistance to copper (Pontel and Soncini 2009). When the cysteine 104 of CueP—previously shown to form a metal-thiolate complex (Abriata et al. 2014)—is replaced by a serine (C104S), this property is lost, confirming that CueP plays an important role in copper adaptation in *Salmonella*.

Then, a thermal profiling assay was used to assess copper’s role in CueP stability. This technique addresses—among other things—the role of metal cofactors in a protein’s resistance to heat-induced denaturation. For instance, copper prevents the thermal destabilisation of the CueO protein, proving that this metal ion is essential for its thermal resilience (Mateus et al. 2020). A WT strain harboring a CueP-STREP expression plasmid was grown until OD 0.5 in the presence of IPTG. The culture was split into two equal volumes and incubated for 30 min with or without 50-µM CuSO₄. Cell extracts were then exposed to increasing temperatures for 3 min, and the soluble proteins were recovered. We observed that CueP was affected in the absence of copper, as illustrated by the thermal destabilisation of the protein above 50°C, whereas the presence of copper allowed CueP to remain stable between 35 and 60°C (Fig. 3C). As a control, we evaluated the amount of RecA in the same samples and observed that copper had no effect on RecA: it aggregates above 50°C, with or without the addition of copper (Fig. 3C). Next, to determine the temperature range in which CueP remained stable, we performed the same experiment as before in the presence of copper and observed bands of identical intensity for CueP between 35 and 90°C, whereas RecA still aggregates above 50°C (Fig. 3D). Finally, to ensure that the decrease in signal intensity is related to aggregate formation, we prepared total extracts without filtration (soluble proteins + aggregates) and found that the intensity of each band was identical for all temperatures tested (Fig. S2). Together, these results point to a role for CueP in copper resistance, which might be mediated by the protein’s ability to bind copper to stabilise its structure.

### The Cpx pathway is activated in the absence of CueO

**Figure 4:**
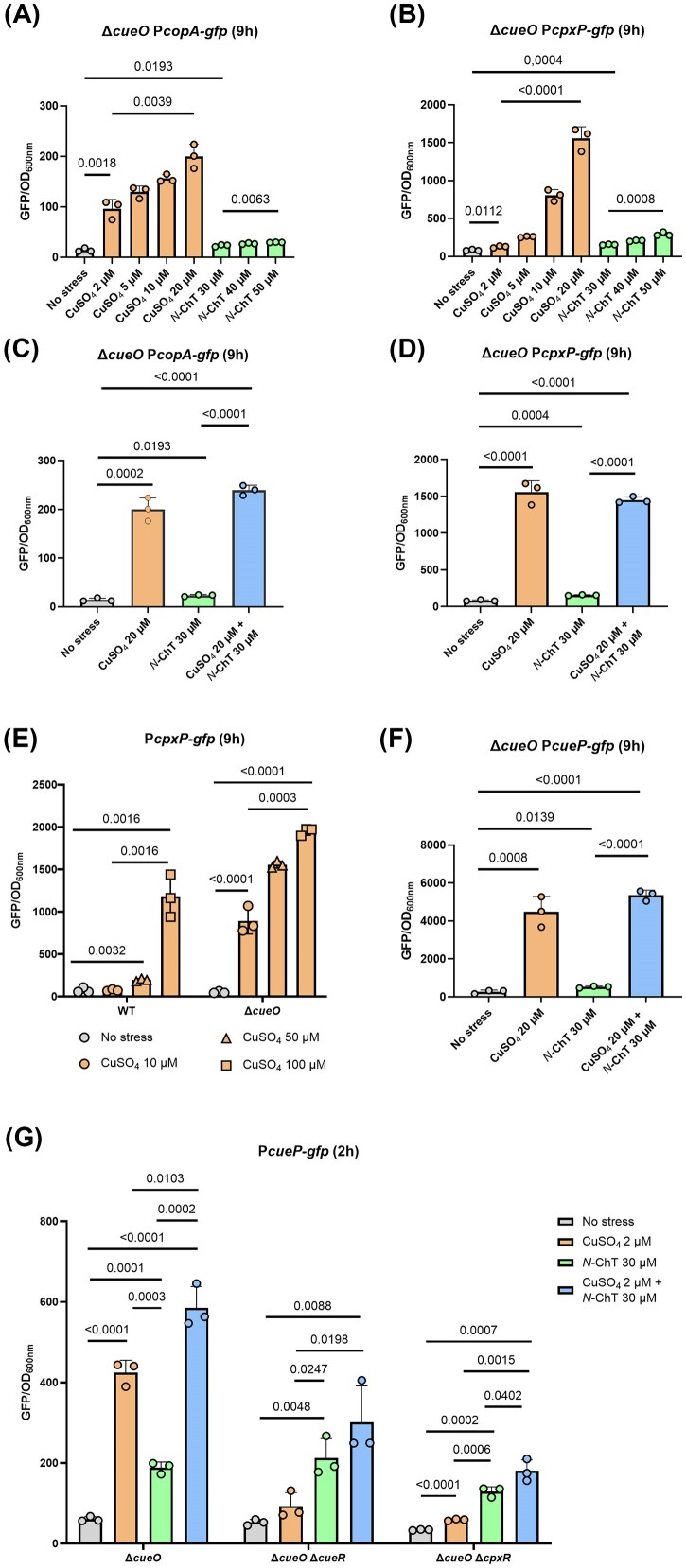
*cueP* expression is activated by copper alone in a Δ*cueO* mutant. A Δ*cueO* mutant strain carrying a P*copA-gfp* fusion (A and C) or a P*cpxP-gfp* fusion (B and D) was grown aerobically in M9 medium. At OD_600_ = 0.3, the strains were subjected to copper stress (orange bars), *N*-ChT stress (green bars), dual copper/*N*-ChT stress (blue bars), or no stress (grey bar). The fluorescence of the fusions was measured using a microplate reader and normalised to the OD_600_ 9 h post-stress. (E) A WT and a Δ*cueO* mutant carrying a P*cpxP-gfp* fusion were grown aerobically in M9 medium. At OD_600_ = 0.3, the strains were subjected to copper stress (orange bars) or no stress (grey bar). The fluorescence of the fusions was measured and normalised to the OD_600_ 9 h post-stress. (F–G) Various strains carrying a P*cueP-gfp* fusion were grown aerobically in M9 medium. At OD_600_ = 0.3, the procedure described previously was followed, and the fluorescence was measured in a Δ*cueO* mutant 9 h post-stress (F), and in a Δ*cueO*, Δ*cueO* Δ*cueR*, and Δ*cueO* Δ*cpxR* mutants 2 h post-stress (G). Results are the means ± standard deviation of three independent experiments. Statistical analysis was performed using Student’s *t*-test. Exact P-values are reported for comparisons with *P* < 0.05 considered statistically significant.

Based on our previous results, we established that *cueP* helps *Salmonella* resist copper and found that this gene’s expression was regulated by both copper and *N*-ChT. This dual regulation led us to question whether *cueP* was actually expressed in our earlier viability experiments, which involved copper but lacked *N*-ChT. We therefore studied the expression of *copA* and *cpxP* in a Δ*cueO* genetic background. We observed a marked increase in fluorescence from the P*copA-gfp* fusion upon exposure to increasing copper doses, whereas the addition of *N*-ChT had no effect (Figs 4A and S3A). Surprisingly, although the P*cpxP*-*gfp* fusion remains induced by *N*-ChT (3-fold) in a Δ*cueO* mutant, it is much more strongly activated by copper alone (15-fold) (Figs 4B and S3B). We then measured the expression level of these two fusions in the presence of copper and *N*-ChT in a Δ*cueO* mutant. Copper alone triggers both fusions at a level comparable to that observed with combined stress (Figs 4C, 4D, S3C, and S3D).

To compare *copA* and *cpxP* expression levels in WT and Δ*cueO* strains, the fluorescence of their respective *gfp* fusions was measured 2 and 9 h post-stress. While the P*copA*-*gfp* fusion exhibited identical expression profiles in both genetic backgrounds (Fig. S3E and S3F), the P*cpxP*-*gfp* fusion was induced 5-fold by 20-µM CuSO_4_ solely in the Δ*cueO* mutant (Fig. S3G & S3H). To determine whether the Cpx pathway responds to copper in a WT background, we monitored P*cpxP*-*gfp* activity across increasing doses of CuSO_4_. A concentration of 100-µM CuSO_4_ was required to achieve an 11-fold (one log) induction in the WT strain, whereas this same level of activation was reached at a 10-fold lower concentration (10-µM) in the Δ*cueO* mutant (Fig. 4E).

**Figure 5:**
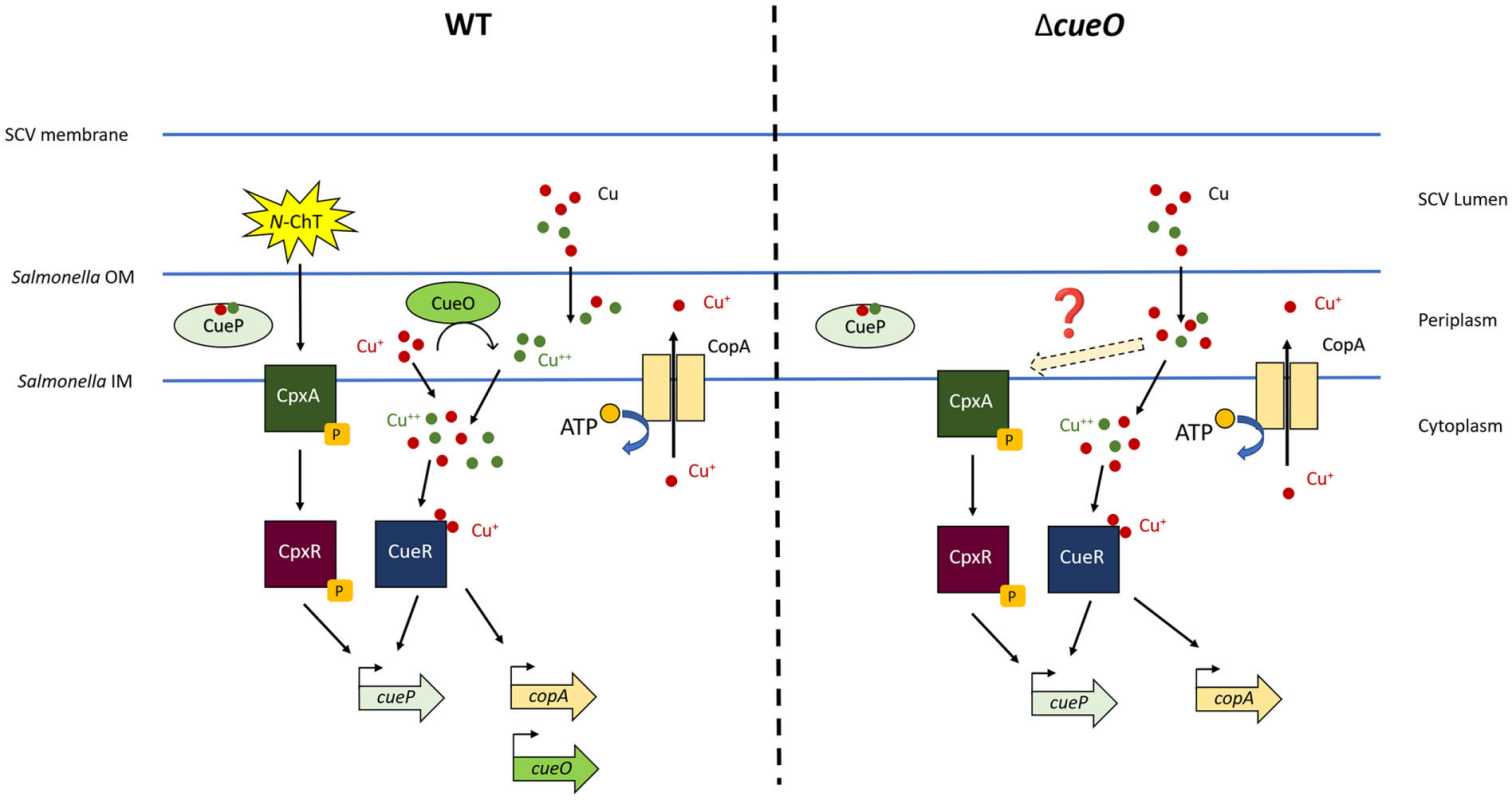
Model for the dual activation of *cueP* by CueR and CpxR in a WT strain and in a Δ*cueO* mutant. *N*-chlorotaurine activates the Cpx pathway when copper simultaneously activates the CueR pathway. Together, these two pathways activate the full expression of the *cueP* gene in a WT strain. In a Δ*cueO* mutant, Cu^+^ is no longer oxidised to Cu^++^, leading to an increase in the periplasmic concentration of Cu^+^, which in turn activates the Cpx and CueR pathways and, ultimately, *cueP* expression.

Finally, we tested the expression of *cueP* in a Δ*cueO* mutant. We observed maximum fusion activation with 20-µM CuSO_4_ and no additive effect with *N*-ChT (Fig. 4F & S4A). This expression profile differs significantly from that observed in a WT strain, where induction of *cueP* by 20-µM CuSO_4_ increases 4-fold, whereas it increases 15-fold in a Δ*cueO* mutant (Fig. S4 B & C). We then wanted to evaluate the role of CueR and CpxR in *cueP* expression in a Δ*cueO* mutant. To this end, we compared the expression of the P*cueP-gfp* fusion in Δ*cueO*, Δ*cueO* Δ*cueR*, and Δ*cueO* Δ*cpxR* mutants. In the latter two strains, *cueP* expression in response to copper was markedly reduced compared to the Δ*cueO* mutant (Fig. 4G), showing that both regulators still play a role in *cueP* activation in this genetic background. It should be noted that the results in Fig. 4(F) were obtained with 2-µM copper (and not 20 µM as in Fig. 4F) because the double mutants Δ*cueO* Δ*cueR* and Δ*cueO* Δ*cpxR* are unable to grow in the presence of 20-µM copper. Taken together, these results show that in the absence of *cueO*, copper alone activates the Cpx and CueR pathways, ultimately allowing full expression of the *cueP* gene (Fig. 5).

## Discussion

In this study, we have completed and refined the sophisticated regulatory architecture governing the expression of *cueP*. We demonstrated that *cueP* transcription is orchestrated by an “AND-like” logic gate integration of two distinct signals: copper and *N*-ChT. We established that while copper specifically triggers the CueR pathway and *N*-ChT activates the Cpx envelope stress response, their simultaneous presence—mimicking the phagosomal environment—leads to maximal *cueP* induction. Furthermore, our results highlight the role of CueP in copper resistance, particularly when the multicopper oxidase CueO is absent, a condition that likely results in the accumulation of toxic Cu^+^ in the periplasm. Finally, we revealed a fail-safe mechanism where, in the absence of CueO, copper-induced envelope stress is sufficient to activate the Cpx pathway, thereby ensuring robust *cueP* expression even in the absence of oxidative signals (Fig. 5).

The simultaneous regulation of *cueP* by metal and oxidative stress represents a remarkable evolutionary adaptation to the intravacuolar lifestyle. During systemic infection, *Salmonella* resides within the *Salmonella*-containing vacuole, where it is bombarded by a combination of host-derived stressors, including the pumping of copper and the generation of ROS and RCS via the oxidative burst. While copper toxicity is generally well managed by resistance mechanisms during the systemic stages of *Salmonella* infection, it presents a distinct challenge during the enteric proliferation phase (Cunrath and Palmer 2021). In the gut environment, *Salmonella* is exposed to naturally high dietary copper concentrations alongside a multitude of harsh conditions—e.g. bile salts, antimicrobial peptides, anoxia—that induce severe bacterial envelope stress. To survive this synergistic assault, *Salmonella* intricately links its metal detoxification strategies with its envelope stress responses. While previous studies have generally examined these stress responses in isolation, our work underscores the importance of studying them in concert. The finding that *cueP* requires both copper (via CueR) and envelope stress signals (via CpxR) for maximal activation suggests that *Salmonella* has evolved in such a way as to restrict the production of this protein to environments where both pathways can be activated. This regulatory strategy differs from the canonical model described in *E. coli*, which relies on the CusCFBA efflux pump for periplasmic copper homeostasis, a system absent in *Salmonella* (Franke et al. 2003). Instead, *Salmonella* appears to have repurposed the Cpx pathway to sense RCS, such as *N*-ChT, and integrate this information with metal sensing. This convergence of signals ensures that the bacterial defense is metabolically efficient, deploying CueP only when the bacterium perceives the specific “fingerprint” of this environment.

Our investigation into the functional role of CueP clarifies its contribution to periplasmic metal homeostasis. While the precise biochemical activity of CueP remains a subject of investigation, our data show that it is required for survival when the primary copper oxidase, CueO, is missing. CueO converts the highly toxic cuprous ion Cu^+^ to the less toxic cupric form Cu^++^ (Singh et al. 2004). Previous studies

In a Δ*cueO* background, the periplasm is presumably enriched with Cu^+^, which is known to cause severe cellular damage by displacing metals from metalloproteins and inducing protein aggregation (Zuily et al. 2022). The fact that *cueP* expression restores copper resistance in this context implies that CueP might handle both Cu^++^ (expected to be the dominant form aerobically and shown to stabilise CueP after heat treatment) and Cu^+^, preventing it from inflicting damage on periplasmic macromolecules. This last observation is consistent with previous work showing that the Δ*cueP* mutant exhibits a copper sensitivity phenotype under anaerobic conditions, where Cu^+^ is dominant (Pezza et al. 2016). This also supports structural studies suggesting CueP binds Cu^+^ with high affinity and cooperates with thioredoxin-like proteins to maintain metal homeostasis (Yoon et al. 2013, Subedi et al. 2019). Therefore, CueP likely acts as a Cu^+^/Cu^++^ sink or chaperone, a function that becomes indispensable under anaerobic conditions or within cellular compartments in which Cu^+^ is prevalent.

A particularly intriguing finding of this work is the activation of the Cpx pathway by copper alone in a Δ*cueO* mutant. A previous study had shown that high concentrations of copper (1 mM) in a rich medium (LB) activated the CueR and Cpx pathways in *Salmonella*. In this study, we used M9 minimal medium and copper concentrations 50 to 500 times lower (2 to 20 µM CuSO₄), which reflect the levels typically found in the phagosome (Wagner et al. 2005). In WT cells, copper levels sufficient to trigger the CueR pathway do not activate the Cpx response. However, the inability to oxidise Cu^+^ in the Δ*cueO* mutant likely leads to Cu^+^-mediated protein misfolding or aggregation in the periplasm, which are classical inducers of the Cpx response. This observation suggests that the Cpx system serves as a secondary, damage-sensing contingency system. If the primary detoxification mechanism (CueO) fails or is overwhelmed, the resulting envelope stress triggers CpxR, which, in turn, boosts *cueP* expression. This regulatory redundancy ensures that *cueP*—which we have shown to be vital in this context—is upregulated even if the specific oxidative signal (*N*-ChT) is absent. This highlights a dynamic interplay between specific metal sensing (CueR) and broad stress sensing (CpxR) to maintain envelope integrity.

Our findings also demonstrate that copper binding is required to maintain the structural integrity of CueP, as evidenced by its rapid thermal destabilisation and aggregation above 50°C in the absence of the metal. The observation that copper supplementation specifically extends CueP stability highlights a direct conformationally stabilising interaction. This metal-dependent structural fortification closely mirrors the behaviour of the *E. coli* CueO, which similarly relies on its copper cofactor to prevent thermal degradation in the cytoplasm (Mateus et al. 2020). Together, these parallel findings suggest a broader paradigm in which copper-handling proteins leverage their cognate metals not merely for function, but as essential structural linchpins. Ultimately, this cofactor-induced stabilisation ensures that CueP remains robustly folded in hostile environments, allowing it to safely sequester excess ions and drive bacterial adaptation during severe copper stress.

All the results of this study advance our understanding of bacterial adaptation by mapping the genetic circuits that allow *Salmonella* to decipher and survive the complex microenvironment of the host immune system. By identifying the dual control of *cueP* by copper and *N*-ChT, we provide a molecular basis for the survival of this pathogen within the phagosome. These findings open several avenues for future research. It will be crucial to validate these regulatory mechanisms *in vivo* to determine the precise kinetics of copper and oxidative stress perception during the course of infection. Furthermore, identifying which specific host cell types (e.g. neutrophils vs. macrophages) trigger this maximal *cueP* response could reveal new aspects of host-pathogen interplay. Finally, advancing toward a better understanding of the biological function of CueP and understanding how it cooperates with other periplasmic chaperones to prevent Cu-induced protein aggregation could inform the development of antimicrobial strategies targeting bacterial stress adaptation.

## Materials and methods

### Strains and microbial techniques

The bacterial strains and plasmids used in this study are listed in Tables 1 and 2, respectively. *Salmonella enterica* serovar Typhimurium strain ATCC 14028 was used as the WT (WT) reference strain. Gene deletions were generated using the one-step λ Red recombination system and subsequently introduced into the WT background via P22 phage transduction (Datsenko and Wanner 2000). Cultures were grown aerobically overnight at 37 °C with shaking in M9 minimal medium supplemented with glucose and casamino acids. Cultures were then diluted 1:100 in fresh medium. When appropriate, antibiotics were added at the following final concentrations for plasmid maintenance: ampicillin (100 µg/mL), chloramphenicol (25 µg/mL), and kanamycin (100 µg/mL).

**Table 1:** Strains used in this study

Strain	Genotype	Source
ATCC14028	*Salmonella enterica* subsp. enterica serotype Typhimurium ATCC14028 (WT)	Laboratory collection
ST711	Δ*cueP*::Cm^r^	This study
ST712	Δ*cueO*::Cm^s^	This study
ST727	Δ*cpxR*::Cm^s^	This study
ST728	Δ*cueR*::Cm^r^	This study
ST735	*cueP*::SPA-tag Kan^r^	This study
ST743	Δ*cueP*::Cm^s^ Δ*cueO*::Cm^r^	This study
ST774	Δ*cueO*::Cm^s^ Δ*cueR*::Cm^r^	This study
ST775	Δ*cueO*::Cm^s^ Δ*cpxR*::Cm^r^	This study

This table contains information regarding the strains used in this study, including strain name, genotype, description, and source.

**Table 2:** Plasmids used in this study

Plasmid	Genotype and description	Source
pJF119EH (empty vector)	P*lac* promoter, IPTG-inducible (Amp^r^)	(Fürste et al. 1986)
p109 (pZEP08)	pBR322 derivative, promoterless *gfp*+ (Cm^r^, Amp^r^, Kan^r^)	(Hautefort, Proença and Hinton 2003)
p197 (P*cpxP-gfp*)	pZEP08 derivative carrying the *cpxP* promoter (Cm^r^, Amp^r^)	This study
p206 (P*copA-gfp*)	pZEP08 derivative carrying the *copA* promoter (Cm^r^, Amp^r^)	This study
p207 (P*cueP-gfp*)	pZEP08 derivative carrying the *cueP* promoter (Cm^r^, Amp^r^)	This study
p208	pJF119EH derivative carrying *cueP* (Amp^r^)	This study
p215	pJF119EH derivative carrying *cueP*—STREP tag (Amp^r^)	This study
p216	pJF119EH derivative carrying *cueP*_C104S_—STREP tag (Amp^r^)	This study

This table contains information regarding the plasmids used in this study, including plasmid name, genotype, description, and source.

The strain ST735 was constructed as follows: the primers 1030 (5’-*cueP*-SPA) and 1031 (3’-*cueP*-SPA-Kan) were used to amplify the SPA-tag Kan^R^ insert from strain DY330 (Loiseau et al. 2017). The insertion of the fragment of interest was checked by PCR. The ST735 strain was generated by transferring the *cueP*-SPA-tag Kan^R^ allele via the standard P22 transduction procedure and was verified by PCR.

### Plasmid construction

The plasmids used in this study are listed in Table 2. The plasmid P*cueP-gfp* was constructed as follows: the primers 1009 (*cueP*-300/*SmaI*) and 1010 (*cueP* Stop/*XbaI*) were used to amplify *cueP* promoter sequence from the genomic DNA of ATCC14028. The PCR product was cloned into pZEP08 using *SmaI* and *Xba*I restriction sites, generating the plasmid P*cueP-gfp*. The same protocol was used to generate the plasmid P*copA-gfp* using the primers 1011 (*copA*-300/*SmaI*) and 1012 (*copA* Stop/*XbaI*). The plasmid P*cpxP-gfp* was constructed in a previous study (Andrieu et al. 2023).

### *N*-chlorotaurine synthesis

*N*-ChT (Cl-HN-CH₂-CH₂-SO₃⁻) was synthesised by mixing equal volumes of 100 mM hypochlorous acid (HOCl; Honeywell) and 100 mM taurine (Sigma-Aldrich) in 0.1 M potassium phosphate buffer (pH 7.4). After a 5-minute incubation at room temperature, the concentration of *N*-ChT was determined by measuring absorbance at 252 nm (ε = 429 M⁻¹ cm⁻¹). The solution was stored at 4 °C until use.

### Fluorescence quantification

Strains harboring P*cpxP-gfp*, P*copA-gfp*, or P*cueP-gfp* reporter plasmids were cultured aerobically in M9-glucose-casamino acids medium supplemented with ampicillin (100 µg/mL) at 37 °C until reaching an OD₆₀₀ of 0.3. 120 µL was transferred to each well of a 96-well flat-bottom black microplate (Greiner Bio-One). To induce stress conditions, 30 µL of *N*-ChT and/or CuSO₄ (Sigma-Aldrich) solutions were added to obtain the desired final concentrations. Fluorescence was measured using a Spark microplate reader (TECAN), with excitation at 482 nm and emission at 515 nm. Fluorescence values were normalised to OD₆₀₀ to account for cell density. Data analysis and graph preparation were performed using GraphPad Prism 8 (GraphPad Software, Inc.). Experiments were conducted over a 10-hour period, and fluorescence values reported correspond to measurements taken at 2 or 9 h post-stress addition. Results are representative of at least five independent biological experiments.

### Immunoblot analysis of CueP production

To determine CueP protein levels following exposure to CuSO₄ and/or *N*-ChT, *S. enterica* cultures were grown aerobically in M9-glucose-casamino acids medium at 37 °C to an OD₆₀₀ of 0.5. Cultures were then exposed to stress for 1 hour. Following treatment, cells were harvested by centrifugation (3000 × g, 5 min), and pellets were resuspended in Laemmli SDS sample buffer. Samples were heated at 95 °C for 10 minutes, separated by SDS-PAGE (12% gel), and transferred to a 0.2-µm nitrocellulose membrane. Immunodetection was performed using anti-FLAG® primary antibody (F1804-200UG, Sigma) diluted 1:10 000 in 5% skim milk, followed by HRP-conjugated anti-mouse IgG secondary antibody (1:10 000). As a loading control, membranes were probed with an anti-RecA antibody (ab63797, Abcam) and HRP-conjugated anti-rabbit IgG, both diluted 1:10 000. Detection was carried out using chemiluminescence with an ImageQuant LAS 4000 system (GE Healthcare). The results shown are representative of at least three independent experiments.

### Copper survival assay

Bacterial strains were grown aerobically at 37 °C with shaking in M9-glucose minimal medium, supplemented with ampicillin (100 µg/mL) and IPTG (100 µM) when using strains carrying CueP-expressing plasmids. When cultures reached an OD₆₀₀ of 0.1, cells were harvested, resuspended in phosphate-buffered saline (PBS), and serially diluted 10-fold. A 5-µL aliquot of each dilution was spotted onto M9-glucose agar plates, supplemented when appropriate with ampicillin (100-µg/mL), IPTG (100 µM), and/or CuSO₄. Plates were incubated at 37 °C for 48 h. Data shown are representative of at least three independent experiments.

### Thermal profiling assay

Thermal profiling experiments were carried out as previously described (Mateus et al. 2020). Briefly, a WT strain harboring a CueP-STREP expression plasmid was grown at 37°C in minimal medium supplemented with ampicillin and 0.1 mM IPTG. At an OD₆₀₀ of 0.5, the culture was split into two equal volumes and incubated for 30 min with or without the addition of 50-µM CuSO₄. Cells were harvested by centrifugation (5 min, 4000 × g, 4°C), washed twice with PBS, and resuspended in PBS to an OD₆₀₀ ∼ 4. Aliquots were subjected to thermal gradients (35–60°C or 35–90°C) for 3 min using a PCR thermocycler. Cells were lysed in PBS supplemented with a protease inhibitor cocktail (Roche), 1 mM MgCl₂, 0.8% NP-40, 50 µg/mL lysozyme, and 50 U/mL benzonase. The extracts were incubated for 20 min at room temperature with shaking, subjected to three freeze-thaw cycles, and centrifuged. Supernatants were filtered through a 0.45-µm 96-well filter plate to remove protein aggregates and mixed with TSTD buffer. Samples were separated by SDS-PAGE (Novex 4–20% gel) and transferred to a 0.2-µm nitrocellulose membrane. Membranes were incubated with an HRP-conjugated anti-STREP antibody (Bio-Rad), and signals were detected using an ECL detection kit. Membranes were reprobed with anti-RecA antibodies, followed by HRP-conjugated anti-rabbit secondary antibodies.

## Supplementary Material

uqag018_Supplemental_File
