# Supplementary material for: Dual regulation of cueP by the CueR and Cpx systems enables Salmonella adaptation to copper and N-chlorotaurine
Source: Microlife. 2026 May 13;7:uqag018. doi: 10.1093/femsml/uqag018 (PMC13201067; doi:10.1093/femsml/uqag018)
Supplement: uqag018_Supplemental_File [file uqag018_supplemental_file.pdf]

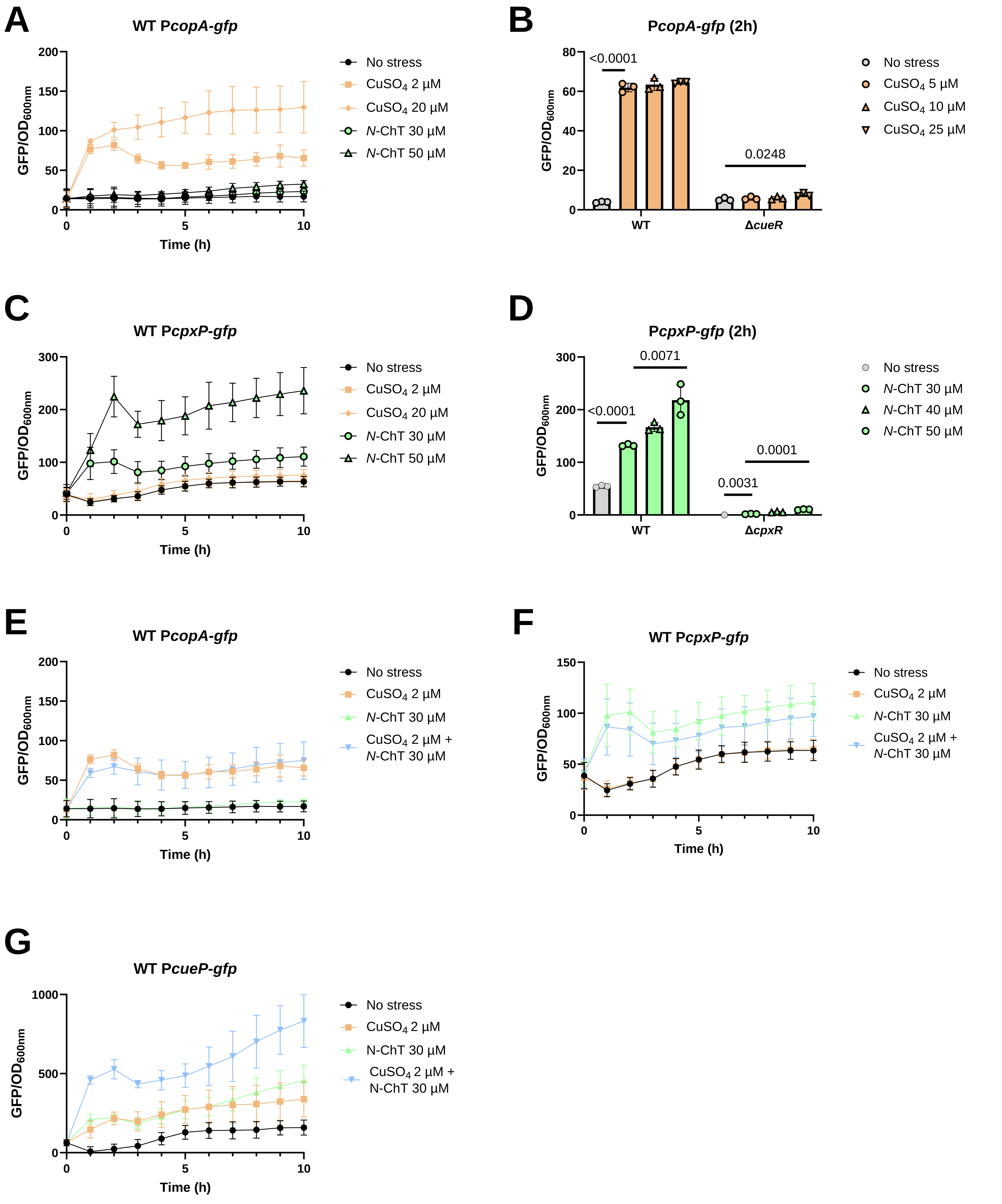

Figure S1: Different *Salmonella enterica* strains carrying a P*copA-gfp* fusion (**A, B** & **E**), a P*cpxP-gfp* fusion (**C, D** & **F**) or a P*cueP-gfp* fusion (**G**) were grown aerobically in M9 medium. At OD_{600} = 0.3, the strains were subjected to copper stress, *N*-ChT stress, dual copper/*N*-ChT stress or no stress (ss indicated). The fluorescence of the fusions was measured using a microplate reader and normalized to the OD_{600} during 10 hours (**A, C, E-G**) or 2 hours post-stress (**B & D**). Results are the means ± standard deviation of at least three independent experiments. Statistical analysis was performed using Student's t test **(B & D)**. Exact P-values are reported for comparisons with $P < 0.05$ considered statistically significant.

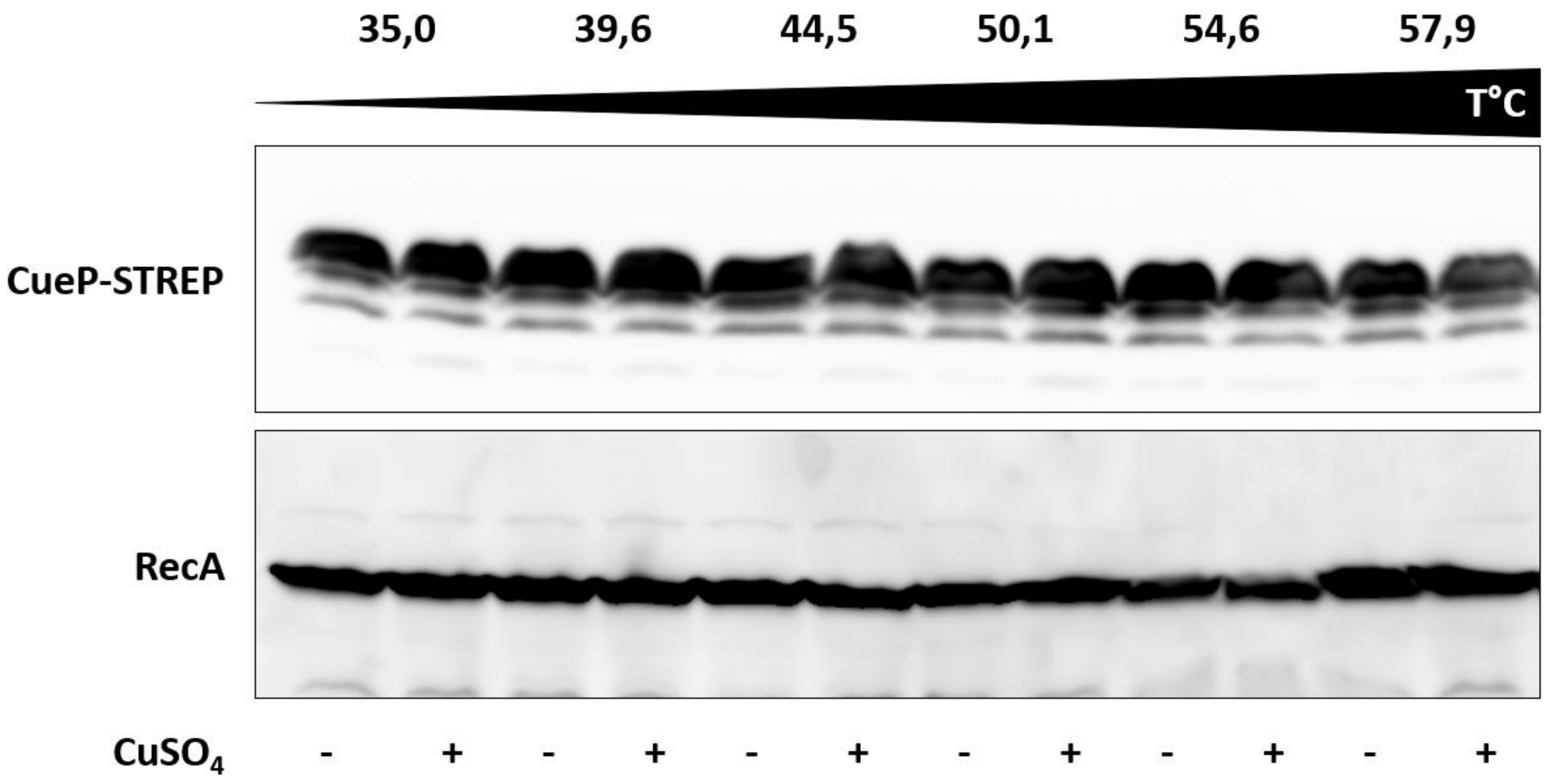

Figure S2: Cellular thermal shift assay of CueP fused to STREP-Tag. Experiments performed in WT living cells with (+) or without (-) 50 µM CuSO4. Aliquots were subjected to thermal gradients from 35 to 60°C (C) during 3 min. Cells were lysed and <u>total fractions</u> were recovered. CueP was detected using HRP-conjugated anti-STREP antibody. As a loading control, run on the same gel, anti-RecA antibodies and HRP-conjugated anti-rabbit secondary antibodies were used.

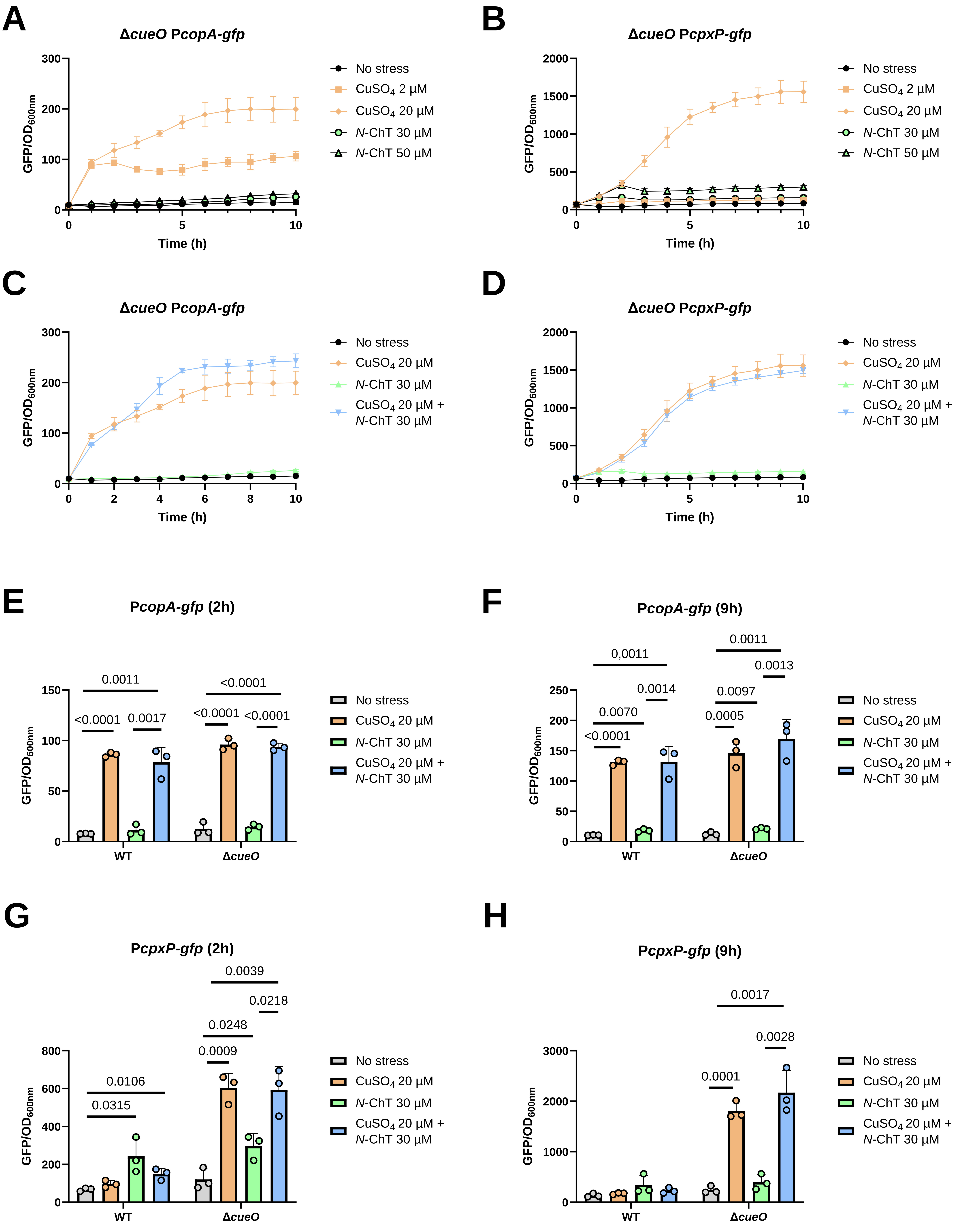

Figure S3: A Δ*cueO* mutant strain carrying a P*copA-gfp* fusion (**A** & **C**) or a P*cpxP-gfp* fusion (**B** & **D**) was grown aerobically in M9 medium. At OD_{600} = 0.3, the strains were subjected to copper stress, *N*-ChT stress, dual copper/*N*-ChT stress or no stress. The fluorescence of the fusions was measured using a microplate reader and normalized to the OD_{600} during 10 hours. (**E-H**) A wild-type strain and a Δ*cueO* mutant carrying a P*copA-gfp* fusion (**E-F**) or a P*cpxP-gfp* fusion (**G-H**) were grown aerobically in M9 medium. At OD_{600} = 0.3, the strains were subjected to copper, *N*-ChT, dual copper/*N*-ChT stress or no stress. The fluorescence of the fusions was measured using a microplate reader and normalized to the OD_{600} 2 and 9 hours post-stress. Results are the means ± standard deviation of at least three independent experiments. Statistical analysis was performed using Student's *t* test (**E-H**). Exact P-values are reported for comparisons with $P < 0.05$ considered statistically significant.

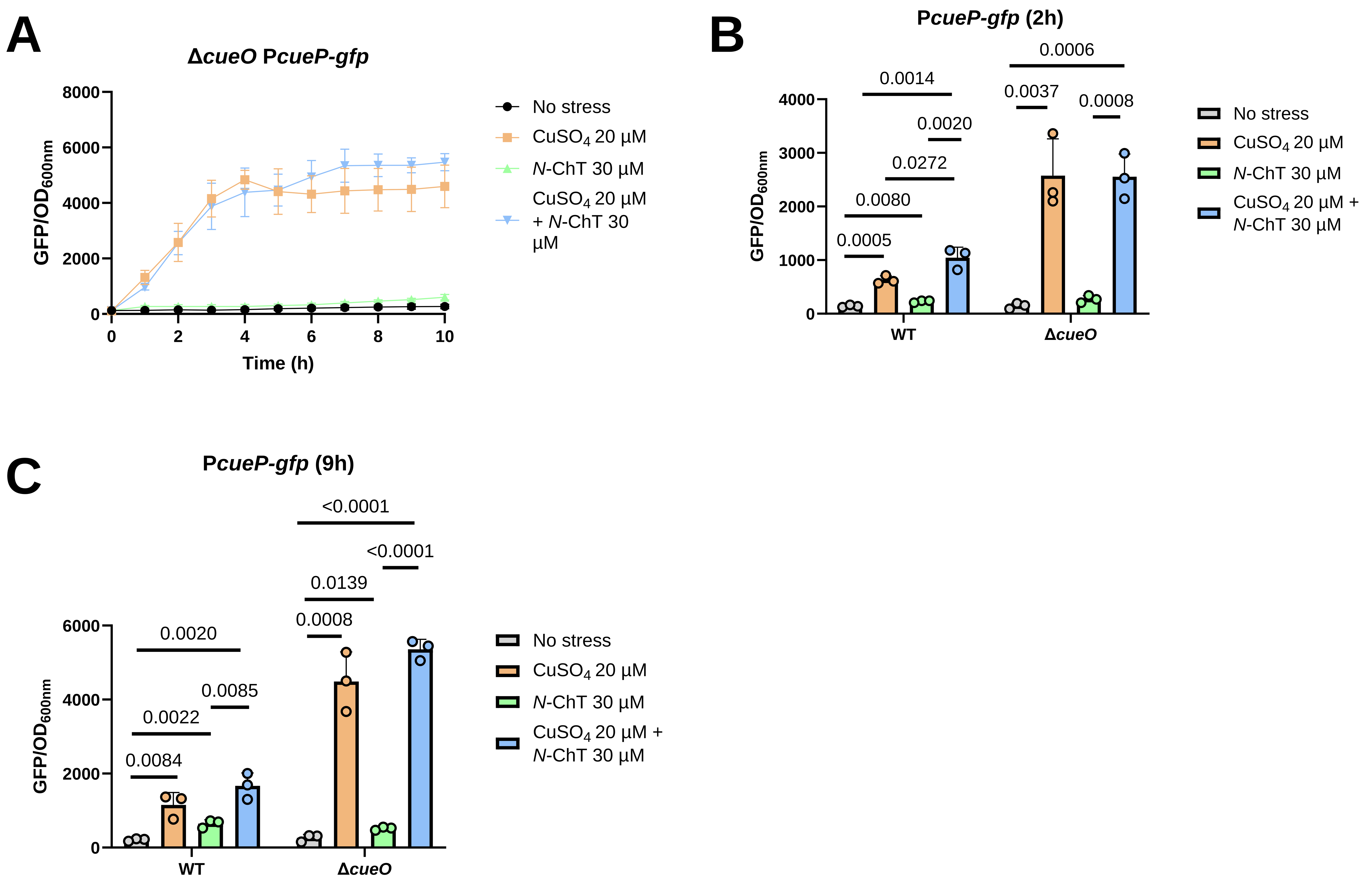

Figure S4: A Δ*cueO* mutant strain carrying a P*cueP-gfp* fusion (**A**) was grown aerobically in M9 medium. At OD_{600} = 0.3, the strain was subjected to copper stress, *N*-ChT stress, dual copper/*N*-ChT stress or no stress. The fluorescence of the fusion was was measured using a microplate reader and normalized to the OD_{600} during 10 hours. (**B-C**) A wild-type strain and a Δ*cueO* mutant carrying a P*cueP-gfp* fusion were grown aerobically in M9 medium. At OD_{600} = 0.3, the strains were subjected to copper, *N*-ChT, dual copper/*N*-ChT stress or no stress. The fluorescence of the fusions was measured using a microplate reader and normalized to the OD_{600} 2 (**B**) and 9 hours (**C**) post-stress. Results are the means ± standard deviation of at least three independent experiments. Statistical analysis was performed using Student's *t* test (**B-C**). Exact P-values are reported for comparisons with P < 0.05 considered statistically significant.
